# Global gene expression analysis of pigeonpea with male sterility conditioned by A_2_ cytoplasm

**DOI:** 10.1002/tpg2.20132

**Published:** 2021-09-08

**Authors:** Abhishek Bohra, Gandam Prasad, Abhishek Rathore, Rachit K Saxena, Satheesh Naik SJ, Shalini Pareek, Rintu Jha, Lekha Pazhamala, Dibendu Datta, Gaurav Pandey, Abha Tiwari, Alok Kumar Maurya, Khela Ram Soren, Mohd Akram, Rajeev K Varshney, Narendra P Singh

**Affiliations:** ^1^ ICAR‐Indian Institute of Pulses Research (ICAR‐IIPR) Kanpur India; ^2^ International Crops Research Institute for the Semi‐Arid Tropics (ICRISAT) Hyderabad India; ^3^ State Agricultural Biotechnology Centre, Centre for Crop and Food Innovation Murdoch University Murdoch Western Australia Australia; ^4^ The UWA Institute of Agriculture The University of Western Australia Perth Australia

## Abstract

Cytoplasmic male sterility(CMS), a maternally inherited trait, provides a promising means to harness yield gains associated with hybrid vigor. In pigeonpea [*Cajanus cajan* (L.) Huth], nine types of sterility‐inducing cytoplasm have been reported, of which A_2_ and A_4_ have been successfully deployed in hybrid breeding. Unfortunately, molecular mechanism of the CMS trait is poorly understood because of limited research invested. More recently, an association between a mitochondrial gene (*nad7*) and A_4_‐CMS has been demonstrated in pigeonpea; however, the mechanism underlying A_2_‐CMS still remains obscure. The current investigation aimed to analyze the differences in A_2_‐CMS line (ICPL 88039A) and its isogenic maintainer line (ICPL 88039B) at transcriptome level using next‐generation sequencing. Gene expression profiling uncovered a set of 505 genes that showed altered expression in response to CMS, of which, 412 genes were upregulated while 93 were downregulated in the fertile maintainer line vs. the CMS line. Further, gene ontology (GO), Kyoto Encyclopedia of Genes and Genomes (KEGG), and protein–protein interaction (PPI) network analyses revealed association of CMS in pigeonpea with four major pathways: glucose and lipid metabolism, ATP production, pollen development and pollen tube growth, and reactive oxygen species (ROS) scavenging. Patterns of digital gene expression were confirmed by quantitative real‐time polymerase chain reaction (qRT‐PCR) of six candidate genes. This study elucidates candidate genes and metabolic pathways having potential associations with pollen development and male sterility in pigeonpea A_2_‐CMS. New insights on molecular mechanism of CMS trait in pigeonpea will be helpful to accelerate heterosis utilization for enhancing productivity gains in pigeonpea.

## Core Ideas

- RNA‐seq was performed on unopened floral buds of isogenic pigeonpea lines.
- A total of 505 differentially expressed genes were identified.
- GO and pathway‐level analysis associated CMS occurrence with four major pathways.
- PPI network analysis revealed a set of 15 hub genes
- The study provides a functional genomics resource to understand male sterility trait in plants.

**Abbreviations**
BP: biological process
CC: cellular component
CMS: cytoplasmic male sterility
DEG: differentially expressed gene
DGE: digital gene expression
GMS: genic male sterility
GO: gene ontology
KEGG: Kyoto Encyclopedia of Genes and Genomes
LFC: log2 fold change
MF: molecular function
PCD: programmed cell death
PPI: protein–protein interaction
qRT‐PCR: quantitative realtime polymerase chain reaction
RNA‐seq: RNA sequencing
ROS: reactive oxygen species
SNP: single nucleotide polymorphism.

## 1 INTRODUCTION

Male sterility systems that circumvent the need of manual emasculation has given a great impetus to crop improvement in agricultural industry via exploitation of hybrid vigour (Dong et al., 2012). In pigeonpea [*Cajanus cajan* (L.) Huth], two types of male sterility systems were discovered: genic male sterility (GMS) and cytoplasmic male sterility (CMS). However, the practical applicability of the GMS system was greatly hampered by its inherent drawback of segregation of a large proportion of fertile plants (Bohra et al., 2020). On the other hand, CMS became popular with the increasing availability of stable male sterile lines, a robust fertility restoration system, and a notable (up to 47%) yield advantage over the check varieties (Saxena et al., 2013). Equally important, availability of male sterility in plants also contributes toward ensuring the seed purity that otherwise remains extremely difficult to achieve through manual emasculation (Liu et al., 2013). To date, nine sterility‐inducing cytoplasms have been reported in pigeonpea that are designated as A_1_–A_9_, a classification relying upon the cytoplasmic donors (Bohra et al., 2016; Saxena et al., 2010). Of these, two cytoplasms, A_2_ and A_4_ derived from *C. scarabaeoides* (L.) Thouars and *C. cajanifolius* (Haines) Maesen, respectively, have been successfully deployed for development of high‐yielding hybrids (Bohra et al., 2020).

Understanding the molecular mechanism of the CMS trait is central to accelerate the progress of hybrid breeding (Mishra & Bohra, 2018). To this end, comparative analysis of four mitochondrial genomes (ICPA 2039, ICPB 2039, ICPH 2433, and ICPW 29) revealed a set of 31 chimeric open reading frames, of which 13 could be associated with A_4_‐CMS (Tuteja et al., 2013). Based on structural variations in mitochondrial genes of CMS and cognate maintainer lines, a 10‐bp deletion in the *nad7a* region was used to develop a diagnostic marker for the detection of A_4_‐CMS in pigeonpea (Sinha et al., 2015a). The study demonstrated that this 10‐bp deletion is specific to A_4_‐CMS and, hence, cannot explain the CMS induced by other cytoplasms. In other words, the molecular mechanism underlying A_2_‐CMS remains elusive, and limited progress has been made, to date, with respect to elucidation of crucial bioprocesses that eventually lead to the generation of nonfunctional pollen grains.

With ever‐increasing throughput and cost‐efficiency, the whole‐transcriptome sequencing, or RNA sequencing (RNA‐seq) has emerged as a promising tool to analyse the expression levels of the genes that participate in regulation of male sterility and fertility restoration in different crops (Huang et al., 2011). The digital gene expression (DGE) approach is increasingly replacing the conventional assays like micro array to study qualitative and quantitative gene expression (Yang et al., 2014). Researchers have conducted comparative transcriptome analysis of flower buds and anthers in CMS and fertile lines in different crops using next‐generation sequencing platforms (Bohra et al., 2016). For instance, differential expression of 365 genes as elucidated by the analysis of the flower bud transcriptomes of the CMS vs. fertile lines with Illumina offered evidence in support of energy deficiency model for CMS induction in soybean [*Glycine max* (L.) Merr.] (Li et al., 2015). Similarly, DGE profiling of male sterile and fertile lines of garlic (*Allium sativum* L.) revealed differential expression for >16,000 genes between the CMS line and fertile line. In this study, the pollen abortion could be credited to impaired respiration and programmed cell death (PCD) in tapetum. In rape (*Brassica napus* L.), the DGE analysis of the self‐pollinating progenies of F_1_ hybrid revealed altered expression of a number of genes, the majority of which were associated with pollen wall formation and carbon and energy metabolism (Yang et al., 2014).

The objective of this study was to examine differential gene expression associated with A_2_–derived CMS in pigeonpea. We used a pair of alloplasmic isogenic lines to elucidate the molecular mechanism of CMS in pigeonpea. The two lines differ only for the fertility trait, which renders them perfectly suitable for transcriptome analysis. Based on comparative transcriptome analysis of flower buds of ICPL 88039A and its cognate maintainer line, ICPL 88039B, we uncovered a set of genes showing altered expression (between the fertile and sterile lines) and the metabolic pathways that are potentially associated with anther and pollen development and CMS induction. Genome‐wide expression patterns are the first to report between isogenic male sterile and fertile lines.

## 2 MATERIALS AND METHODS

### 2.1 Sample preparation and sequencing

Total RNA was extracted from the flower bud tissue of ICPL 88039A and ICPL 88039B using the TRIzol kit (Invitrogen). The CMS line ICPL 88039A was developed by Bohra et al. (2017) through standard backcross procedure. A popular early pigeonpea variety, ICPL 88039, was crossed as pollen parent with CMS line GT 288A (seed parent). The male sterile F_1_ was crossed with the recurrent parent ICPL 88039 to generate BC_1_F_1_ plants. The male sterile BC_1_F_1_ plant was again crossed with ICPL 88039. The scheme was followed till BC_6_F_1_ to obtain a stable CMS line with 99% recovery of the nuclear genome of ICPL 88039. During the backcrosses, the self‐pollinated plant of ICPL 88039 (the same that was used for crossing) served as maintainer. The resulting CMS and the corresponding maintainer line were designated as ICPL 88039A and ICPL 88039B, respectively. The CMS line ICPL 88039A and maintainer line ICPL 88039B were isogenic except for anther color, which reflected the formation of nonfunctional or functional pollen. Both lines are semispreading and indeterminate in growth habit with plants having narrowly oblong leaves, yellow flowers, immature pods with green color, and purple streaks and pods devoid of pubescence (Bohra et al., 2017).

**FIGURE 1:**
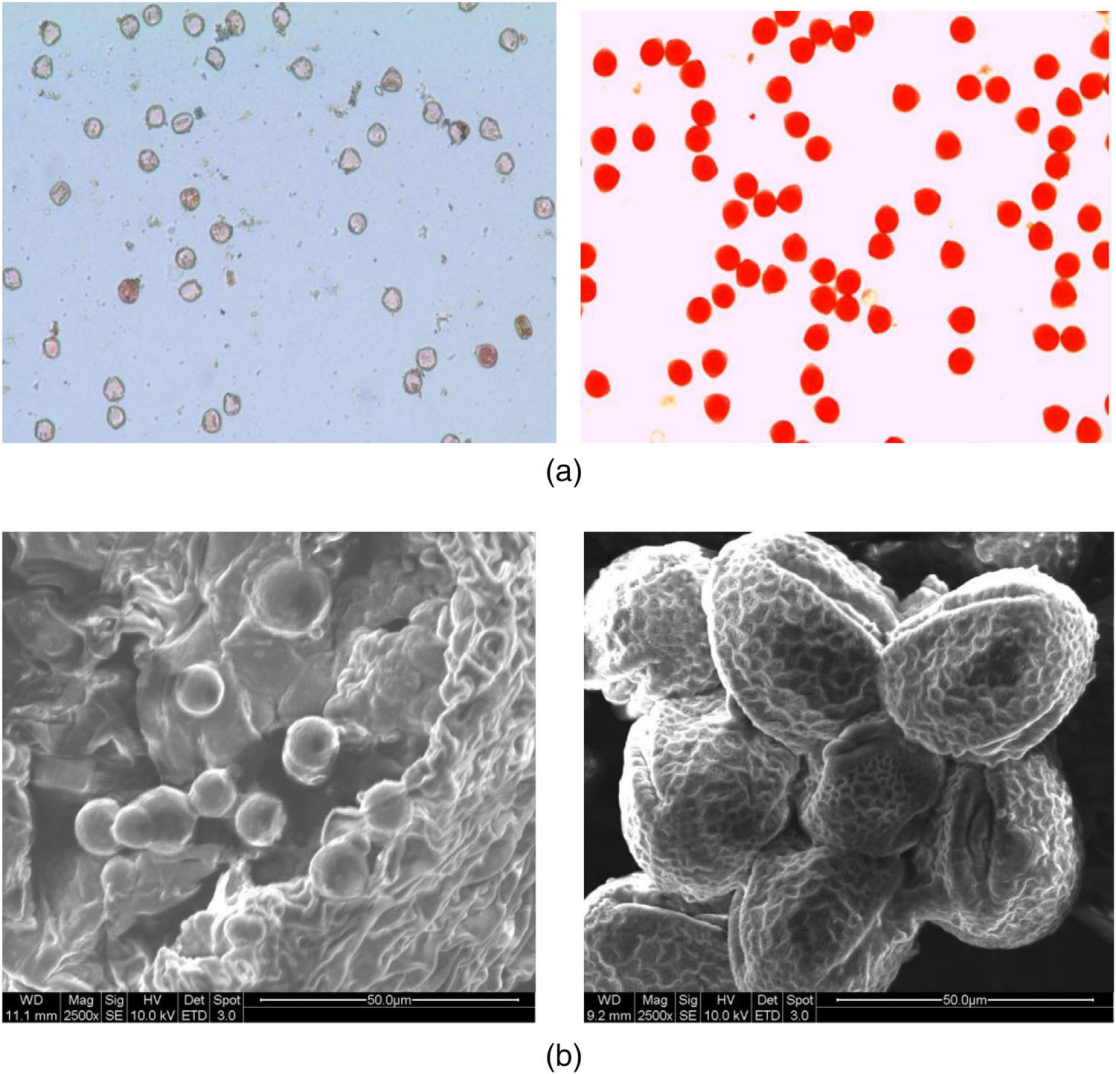
(a) Pollen grains as visualized with 1% acetocarmine. Pollen grains from cytoplasmic male sterility line stained poorly (left), while the fertile pollen grain stained dark red colored (right) (b) Scanning electron microscopy (SEM) analysis of the pollen grains from ICPL 88039A (left) and ICPL 88039B (right), Scale bar, 50 μm

The pollen fertility assay of CMS and fertile lines are shown in Figure 1. The integrity of total RNA was checked by 1% agarose gel electrophoresis; the concentration and purity were determined using NanoDrop (Thermo Scientific) and Agilent 2100 Bioanalyzer. Three biological replicates per sample were used for sequencing. Pair‐end libraries were prepared using Illumina TruSeq stranded mRNA library preparation kit as per its described protocol. Cluster generation and sequencing were performed using Illumina platform following Das and Majumder (2019). The read length of the sequencing was 2 by 150 bp.

### 2.2 Variant calling and identification of DEGs from the RNA‐seq data

Sequencing reads in the fastq format were quality checked using FastQC v0.11.8 tool (Andrews, 2010). Then Illumina TruSeq adapters were trimmed and low‐quality reads were discarded using Trimmomatic v0.36 by specifying parameters SLIDINGWINDOW:4:15 and minimum read length cutoff of 35 bp (Bolger et al., 2014). The clean reads in FASTQ format were aligned to the reference genome using STAR v2.7.1a aligner (Dobin et al., 2012). The SNPs were called using GATK pipeline and SNPs were subsequently filtered based on minimum base quality (minQ < 30) and minimum depth (minDP < 5) (McKenna et al., 2010). All the identified SNPs were annotated, and unique SNPs in each sample were listed. The SNP density ratio per 1000 bp per gene was calculated and plotted on 11 chromosomes using Circos tool (Krzywinski et al., 2009). The high‐quality reads were de novo assembled using Trinity v2.8.4 (Grabherr et al., 2011). Each of the six high‐quality fastq paired‐read files (three biological replicates of each ICPL 88039A and ICPL 88039B) were aligned to the de novo transcriptome assembly using Bowtie v2.3.5.1 (Langmead & Salzberg, 2012) and RSEM was used to estimate expression values based on the alignment (Li & Dewey, 2011). The differentially expressed genes (DEGs) between CMS line and maintainer line were identified using DESeq2 R package (Love et al., 2014). The significant DEGs for further analysis were filtered with adjusted *p* value < .1 (observed highest *p* value is .003) and log_2_ fold change (LFC) ≥ 1. EnhancedVolcano R package was used to generate volcano plots (Blighe et al., 2019).

**Core Ideas**

- RNA‐seq was performed on unopened floral buds of isogenic pigeonpea lines.
- A total of 505 differentially expressed genes were identified.
- GO and pathway‐level analysis associated CMS occurrence with four major pathways.
- PPI network analysis revealed a set of 15 hub genes.
- The study provides a functional genomics resource to understand male sterility trait in plants.

### 2.3 Annotation and functional characterization of DEGs

To find the gene annotations, DEGs were handled as queries and BLASTx search was performed against pigeonpea protein sequences with a significant threshold e‐value of <0.00001 (Camacho et al., 2009). Gene ontology (GO) annotations for the identified DEGs were taken from the annotations given in the original pigeonpea genome assembly files and LegumeIPv2 database (Li & Dewey, 2011). Further, DEGs were subjected to GO enrichment analysis to find enriched molecular functions, biological process, and cellular component using topGo R package (Alexa & Rahnenfuhrer, 2019) and BiNGO v3.0.3 (Maere et al., 2005) plugin in Cytoscape v3.7.1 (Shannon et al., 2003). Two statistical tests, namely Kolmogorov–Smirnov test and Fisher's exact test, were used to select the top 15 enriched pathways on the basis of *q* value (the lesser the *q* value the more significant is the pathway) for both up‐ and downregulated DEGs. The Kyoto Encyclopedia of Genes and Genomes (KEGG) Orthology‐Based Annotation System (KOBAS v3.0) was used to calculate the enriched KEGG pathways from the DEGs (Xie et al., 2011). Furthermore, high‐throughput data visualization software MapMan v3.6.0RC1 was used for pathway visualization (Schwacke et al., 2019). To generate the mapping file, pigeonpea CDS sequences were assigned to the most recent MapMan ontology using Mercator4 v2.0.

### 2.4 Construction of protein–protein interaction networks

The protein‐protein interaction (PPI) network was built using web‐based tool STRING v11.1 based on the orthologs of identified DEGs (http://string-db.org; Szklarczyk et al., 2018). This database generates a PPI network based on coexpression, interaction sources, experiments, and literature mining of query proteins and genes. An interaction score of >0.2 was applied. The PPI network was visualized on Cytoscape v3.7.1.

### 2.5 Quantitative real‐time PCR

The quantitative real‐time polymerase chain reaction (qRT‐PCR) analysis was employed to validate the gene expression patterns inferred from RNA‐seq data. Total RNA from flower bud tissue of ICPL 88039A and ICPL 88039B was isolated using the method of Jorge and Omar (2011) followed by DNase I (Thermo Fisher) treatment. The integrity of isolated RNA was checked on 1% agarose formaldehyde (FA) gel electrophoresis. The concentration of each sample was checked on the BioPhotometer plus (Eppendorf) and three micrograms of RNA was used for first‐strand cDNA synthesis using the high‐capacity cDNA reverse transcription kit (Thermo Fisher,) following the manufacturer's protocol. The qRT‐PCR was carried out using PowerUpTM SYBR Green Master Mix (Applied Biosystems) reaction on a QuantStudioTM 5 Real‐Time PCR System (Applied Biosystems) according to the manufacturer's instructions. The PCR conditions for all the qRT‐PCR reactions were used as follows: 2 min at 50 °C, 2 min at 95 °C, and 40 cycles of 15 s at 95 °C, 15 sec at 60 °C, and 1 min at 72 °C. Actin 1 (Sinha et al., 2015b) was used as internal control. Each reaction was performed in three biological and three technical replicates along with no template control. Relative quantification (RQ) was determined by the following formula: RQ = 2^−ΔΔCt^ (Livak & Schmittgen, 2001).

## 3 RESULTS

### 3.1 Transcriptome sequencing and de novo assembly

To obtain genes responsible for CMS induction in ICPL 88039A, total RNA was isolated from CMS line and cognate fertile line (ICPL 88039B) for the generation of cDNA library. We obtained 50949127 and 69870800 clean paired‐end reads in the case of ICPL 88039A and ICPL 88039B, respectively, following trimming of raw reads. Average length of clean reads was 130.48 bp. The clean reads from each sample were aligned to the de novo transcriptome assembly with an overall alignment rate ranging from 75.28 to 81.47%. In the de novo assembly, we found an average contig length of 566.57 bp and an N50 of 994 bp. The average percentage of GC content in the assembly was 40.56%. This transcriptome shotgun assembly project has been deposited at NCBI GenBank under the accession GIME01000000.

### 3.2 Identification of SNPs, DEGs, and GO annotations

**FIGURE 2:**
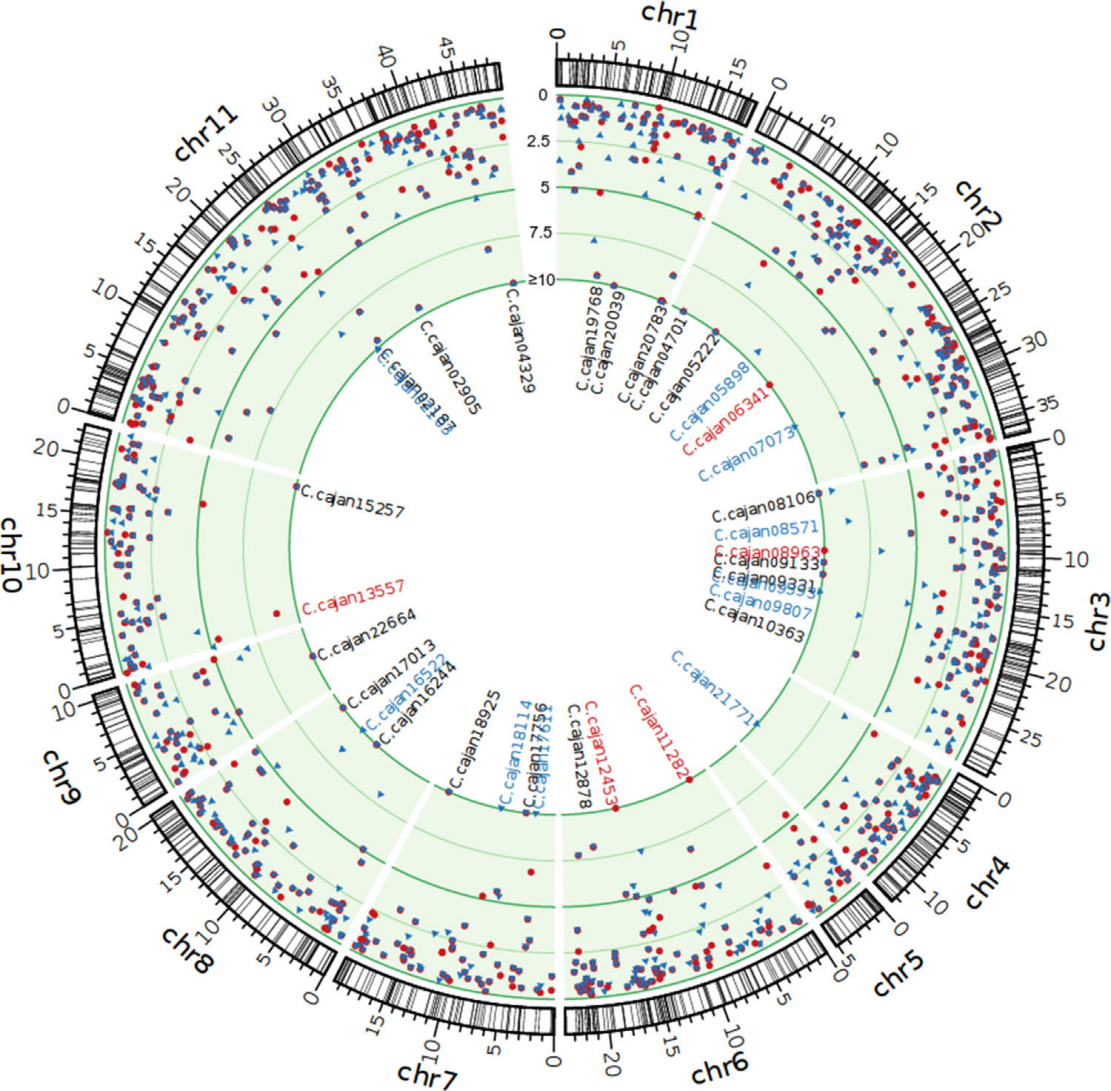
Single nucleotide polymorphism (SNP) density ratios of genes from ICPL 88039A and ICPL 88039B. The SNP density ratios of genes from ICPL 88039A and ICPL 88039B are shown as red circles and blue triangles, respectively based on their SNP density ratio score (0 to ≥10). Genes with higher SNP density ratio from ICPL88039A and ICPL88039B were labeled as red and blue, respectively, whereas genes having higher SNP density in both lines were labeled as black in the center of the plot

**FIGURE 3:**
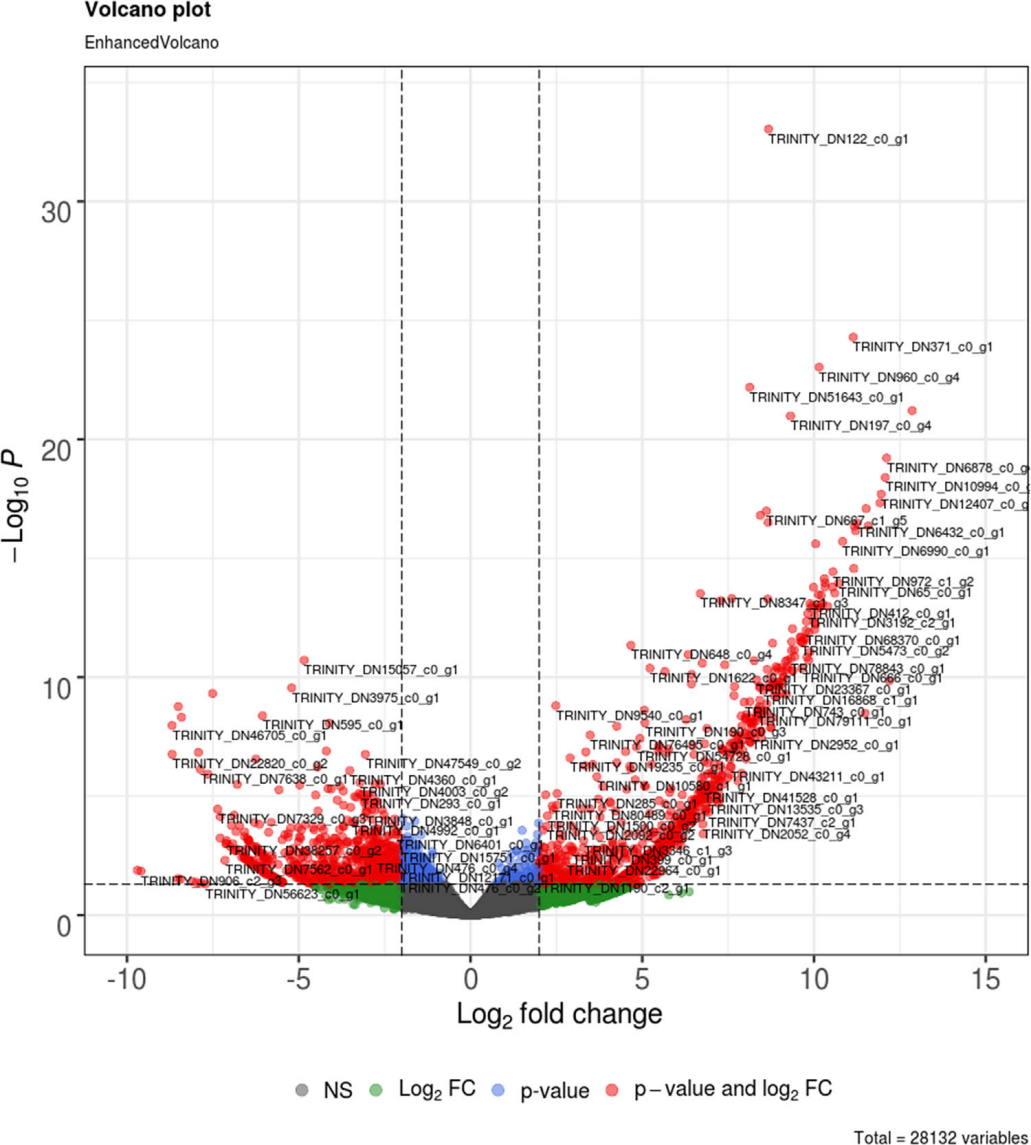
Volcano plots showing expression abundance of transcripts between ICPL 88039A and ICPL 88039B. Transcripts with upregulated expression in ICPL 88039B are shown toward the right, while downregulated transcripts in ICPL 88039B are toward the left. The most statistically significant transcripts are toward the top represented by red dots and slightly transcripts genes are shown by green dots

**FIGURE 4:**
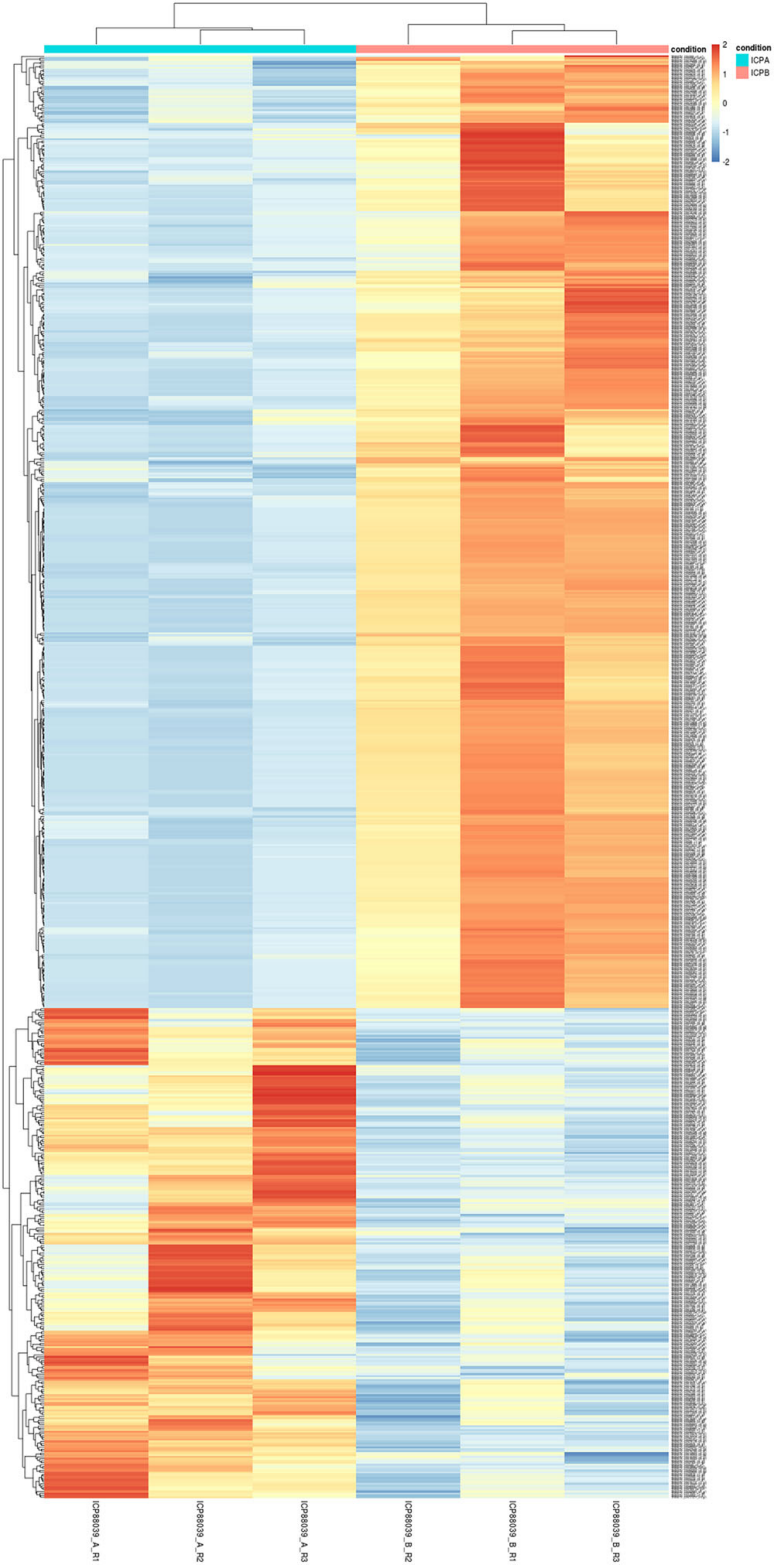
Hierarchical cluster analysis of differentially expressed genes of the genotypes ICPL 88039A and ICPL 88039B

Variant calling using GATK identified a total of 5,331 SNPs, of which 1,112 and 2,007 were unique to ICPL 88039A (three replicates merged) and ICPL88039 B (three replicates merged) lines, respectively and their annotations were also obtained. Figure 2 illustrates the SNP density ratios plotted on 11 chromosomes. The SNPs were found in 1,528 and 1,860 genes in ICPL 88039A and ICPL 88039B, respectively (Supplemental Table S1). Transcript quantification from the RNA‐seq data was performed with RSEM, and DESeq2 was used for differential expression analysis of the estimated counts (Figures 3 and 4). Using CMS line as a reference, a total of 505 DEGs were obtained using *p* value of <.003 and absolute LFC value of ≥1 as the threshold. Of these DEGs, 412 showed higher expression in male fertile line with LFC ranging from 1.20 to 12.8, whereas 93 had lower expression with LFC varying between −1.10 and −8.6 (Supplemental Table S2). To better analyze variability among replicates, all six samples were plotted in a two‐dimensional space using PCA, and the resulting PC1 and PC2 explained 34 and 25% of the variation, respectively (Supplemental Figure S1). Similarly, a high degree of biological replicate reproducibility in the present study could be visualized from sample distance matrix (Supplemental Figure S2).

Gene ontology terms enriched with *p* value <.05 were taken into consideration and the DEGs were annotated to functional categories. In case of the 93 downregulated DEGs in ICPL 88039B, 127 GO terms were enriched. The 127 GO terms belonged to 71 biological process (BP), 24 molecular function (MF), and 32 cellular component (CC) categories. Similarly, 242 GO terms corresponding to 116 BP, 90 MF, and 36 CC were enriched in case of upregulated DEGs.

### 3.3 TopGO annotations and network representations of DEGs

**FIGURE 5:**
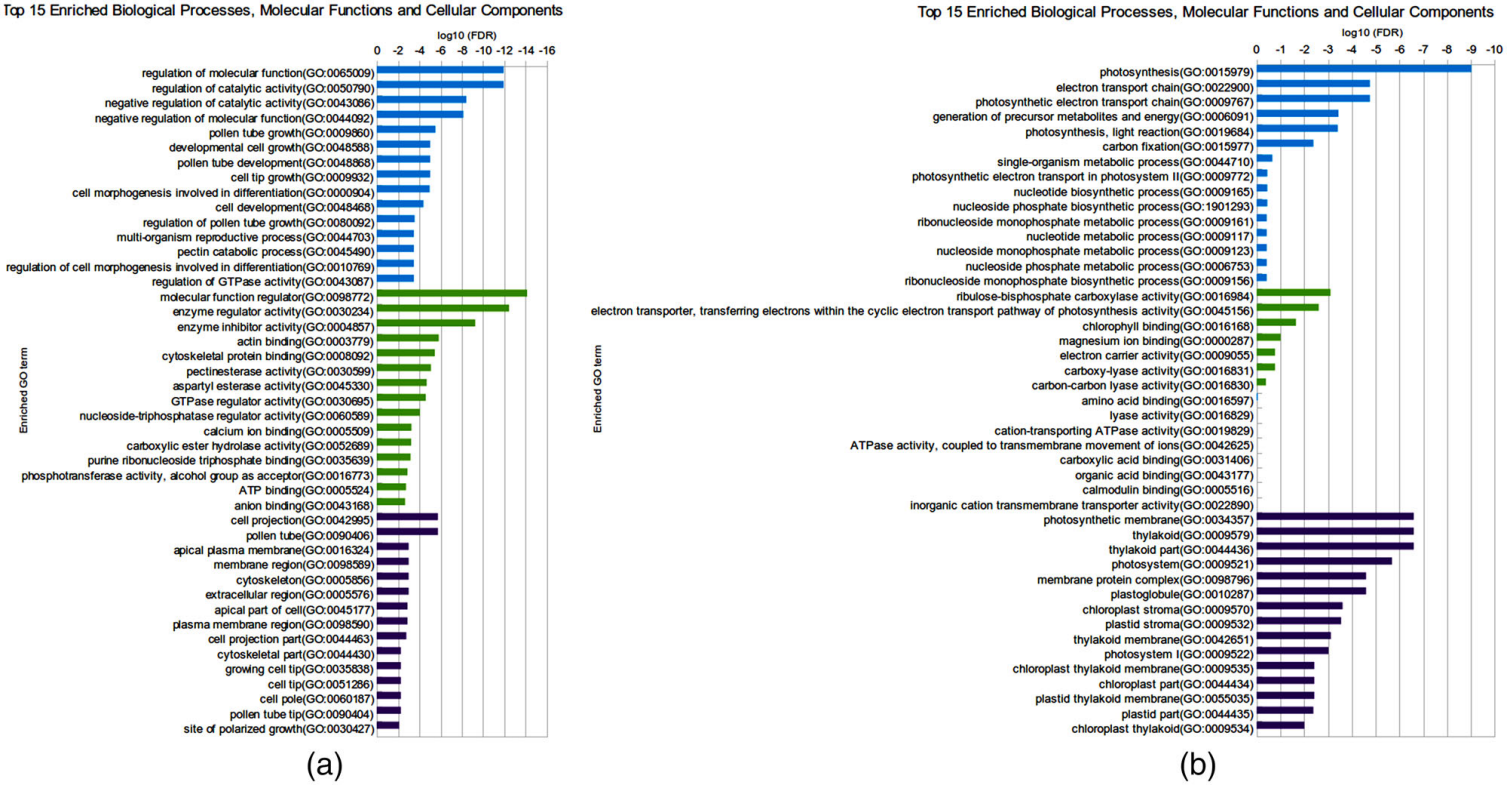
Top 15 enriched gene ontology (GO) terms in biological process (BP), molecular function (MF), and cellular component (CC) categories. (a) Upregulated differentially expressed genes (DEGs) in ICPL 88039B. (b) Downregulated DEGs in ICPL 88039B

TopGO analysis was used to analyze DEGs for their association with the dominant pathways (Supplemental Table S3). Figure 5a depicts topmost enriched terms regulation of molecular function (GO:0065009), molecular function regulator (GO:0098772), and cell projection (GO:0042995) in BP, MF, and CC, respectively, in the case of upregulated genes in fertile line. The other functional groups among BP category were regulation of catalytic activity, pollen tube growth, developmental cell growth, and pollen tube development. Similarly, enzyme regulator activity and enzyme inhibitor activity were significantly enriched among MF category, whereas pollen tube, apical plasma membrane, membrane region, and cytoskeleton were significantly enriched among CC category.

Among the downregulated genes, photosynthesis (GO:0015979), ribulose‐bisphosphate carboxylase activity (GO:0016984), and photosynthetic membrane (GO:0034357) were the most dominant terms in BP, MF and CC categories, respectively, followed by electron transport chain (GO:0022900), electron transporter (GO:0045156), and thylakoid (GO:0009579) (Figure 5b). The network between the associated pathways was constructed and the major top GO terms were identified (Supplemental Table S4). Representative images of the major GO terms of upregulated genes associated with CC category are presented in Supplemental Figures 3a and 3b.

### 3.4 Pathway analysis and functional classification of DEGs

**FIGURE 6:**
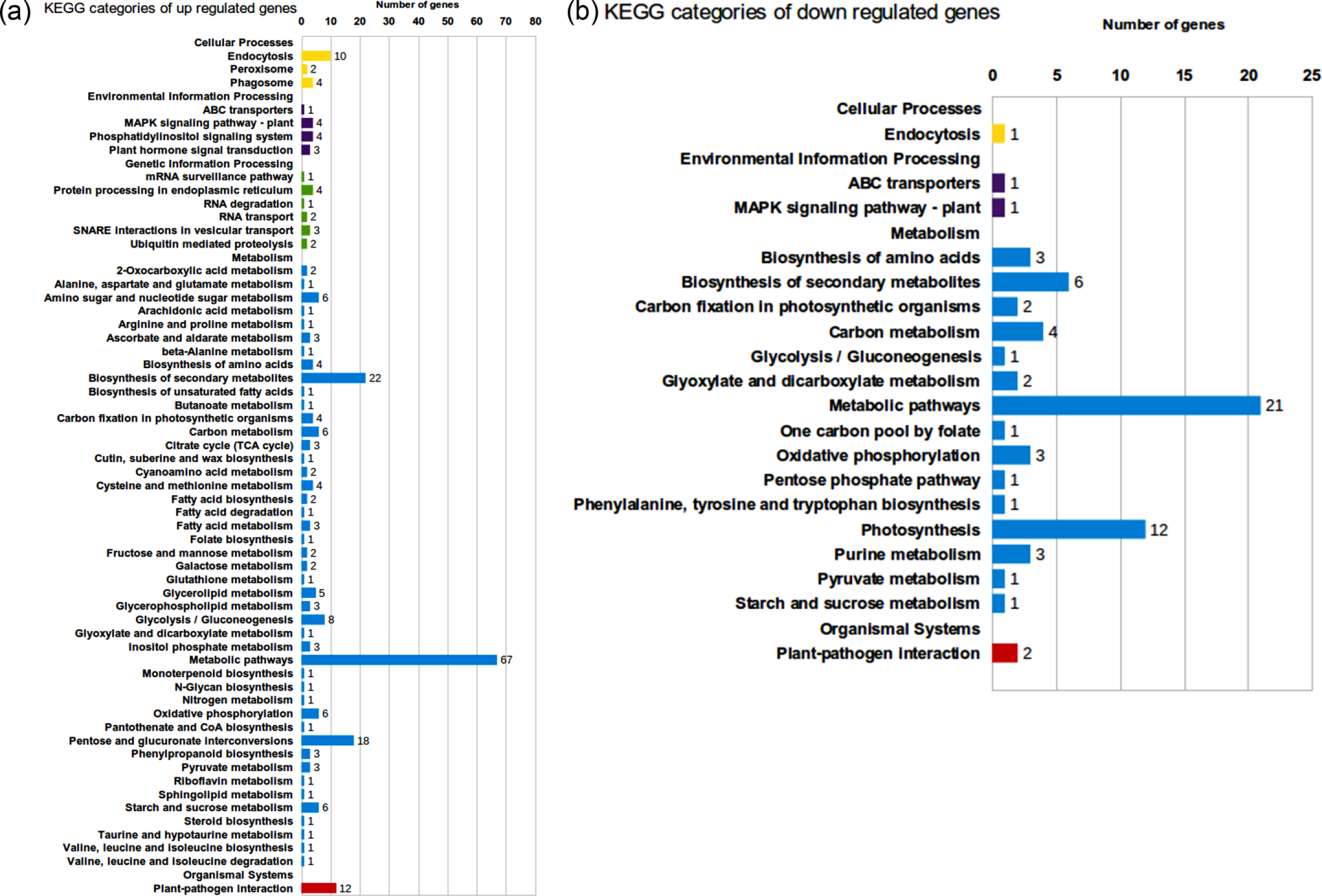
The best represented Kyoto Encyclopedia of Genes and Genomes (KEGG) pathways of (a) upregulated differentially expressed genes (DEGs) and (b) downregulated DEGs in ICPL 88039B

**FIGURE 7:**
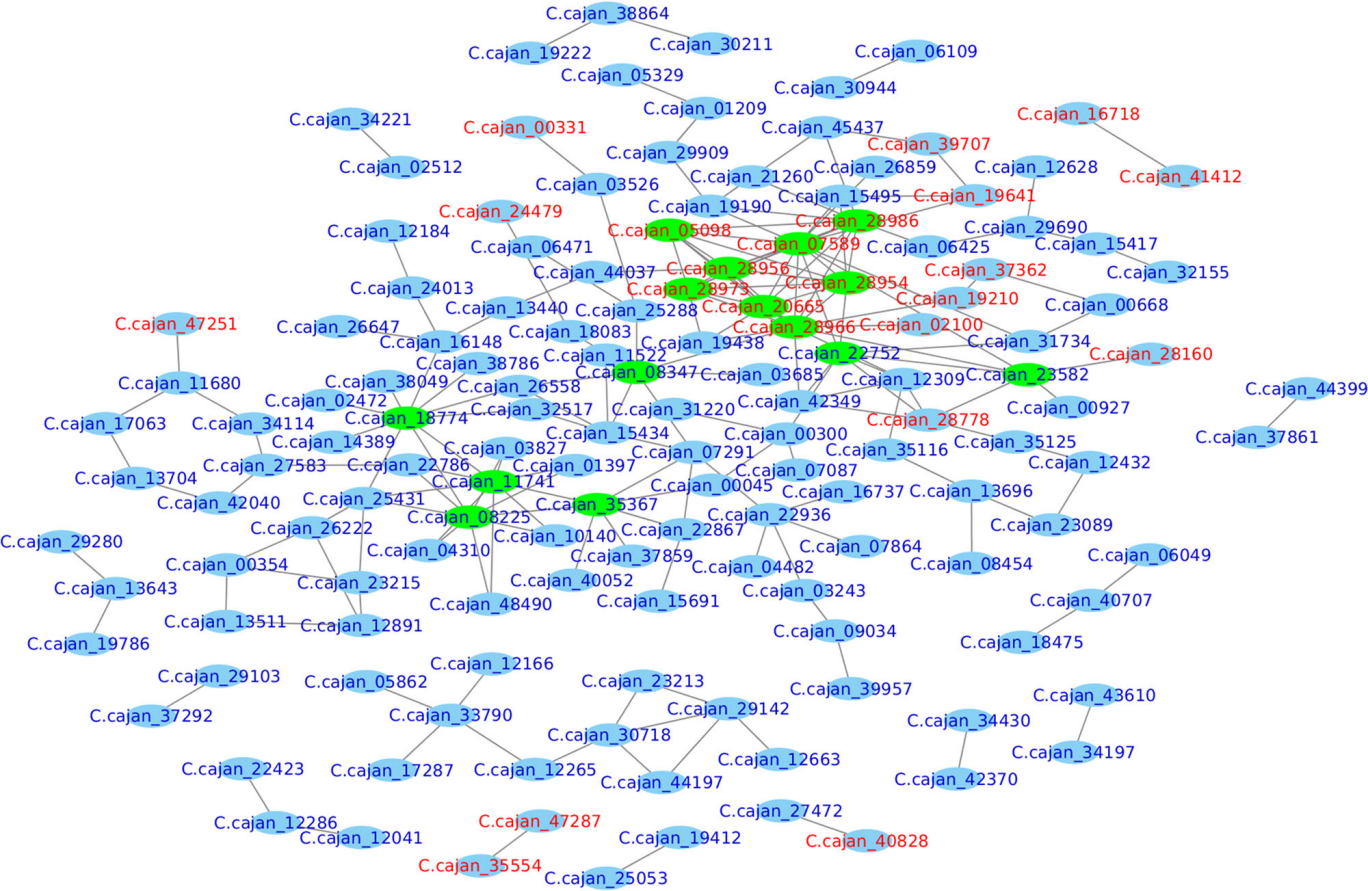
Protein–protein interaction networks between DEGs.The up‐ and downregulated DEGs of ICPL 88039B are shown in blue and red color, respectively. Top 15 nodes having high degree and number of directed edges are highlighted in green color

Analysis using KEGG pathway enrichment was conducted to elucidated the involvement of DEGs to various pathways (Supplemental Table S5). In total, 261 and 67 DEGs from upregulated and downregulated categories were assigned to 59 and 19 KEGG pathways, respectively. As shown in Figures 6a and 6b, the pathways with the highest representation from the upregulated genes were metabolic pathways (KEGG ID: ccaj01100, 67) followed by biosynthesis of secondary metabolites (KEGG ID: ccaj01110, 22), pentose and glucuronate interconversions (KEGG ID: ccaj00040, 18), and plant‐pathogen interaction (KEGG ID: ccaj04626, 12). A representative image shows DEGs affecting the KEGG pathway pentose and glucuronate interconversions (Supplemental Figure S4). Other enriched pathways included glycolysis and gluconeogenesis (KEGG ID: ccaj00010, 8), carbon metabolism (KEGG ID: ccaj01200, 6), starch and sucrose metabolism (KEGG ID: ccaj00500, 5), glycerolipid metabolism (KEGG ID: ccaj00561, 5), carbon fixation in photosynthetic organisms (KEGG ID: ccaj00710, 5), amino sugar and nucleotide sugar metabolism (KEGG ID: ccaj00520, 5), protein processing in endoplasmic reticulum (KEGG ID: ccaj04141, 5), etc. Metabolic pathways was also the most representative pathway in the case of downregulated genes followed by photosynthesis (KEGG ID: ccaj00195, 14), Biosynthesis of secondary metabolites (KEGG ID: ccaj01110, 6), and carbon metabolism” (KEGG ID: ccaj01200, 4). Pathway analysis using MapMan showed that several DEGs were involved in cell wall organization, lipid, and metabolism (Supplemental Figure S5). Furthermore, the PPI network analysis was performed on DEGs in order to find potential functional interactions between them. The PPI network analysis revealed a set of 15 hub genes having high degree and number of directed edges (Figure 7). These hub genes are involved in various biological processes including ATP synthesis, pectin catabolic process, carbohydrate metabolic process, and photorespiration (Supplemental Table S6).

### 3.5 DEGs potentially associated with the CMS

Carbon and energy metabolism is one of the key pathways that involved altered expression of 11 genes in glycolysis and TCA and six genes in oxidative phosphorylation (Supplemental Table S5). Among these, glyceraldehyde‐3‐phosphate dehydrogenase (*C.cajan_23582*), malate dehyrogenase (*C.cajan_*22752), isocitrate dehydrogenase (*C.cajan_31734*), and fructokinase (*C.cajan_07087*) were downregulated in the CMS line, whereas pyruvate kinase (*C.cajan_28778*) was upregulated. Likewise, genes encoding proton‐transporting ATPase 6 (*C.cajan_45656*), V‐ATPase and ATPase 9 (*C.cajan_45437, C.cajan_21260*, and *C.cajan_37897*), and pyrophosphate phosphohydrolase (*C.cajan_19190*) had lower expression in the CMS line.

Three and five DEGs involved in ascorbate and aldarate metabolism and starch and sucrose biosynthesis, respectively, showed higher expression levels in male fertile line, and these genes are known to control the cell division and maintain the carbon flux in the sink tissues during pollen development. SUC2, a sucrose transporter protein (*C.cajan_35396*) responsible for the transport of sucrose into the cell and tissues, LEA D‐7, a late embryogenesis protein (*C.cajan_06049*), LEA‐14 (*C.cajan_34939*), and LEA‐D 34 (*C.cajan_06906*) expressed low in CMS lines vs. its isogenic fertile line.

We obtained 33 DEGs that participate in pollen‐related processes (Supplemental Table S2). Six DEGs underexpressed in the CMS line including receptor‐like protein kinase (*C.cajan_10753*) that control pollen tube behavior through timely rupturing to release germ cells. Similarly, low expression levels were recorded in the CMS line for two genes responsible for pollen tip growth and three genes involved in pollen wall assembly. Lower expression was also recorded in the CMS line for Rho‐guanine exchange factor (*C.cajan_00354*), which acts downstream to PRK2 in order to control the polarization of pollen tube growth and the gene *C.cajan_04138* implicated in glucan synthase activity associated with normal development of male gametophyte. These genes showing downregulation in ICPL 88039A are known to control several functions including formation of pollen exine, callose wall, and pollen tube growth.

Differentially expressed genes also included *C.cajan_44741*, *C.cajan_04312, C.cajan_27022*, *C.cajan_18474*, and *C.cajan_32517* coding for pectate lyase 3, polygalactouronase, glycan metabolism, pollen‐specific SF3, and formin‐like protein 5, respectively, that are known to play a significant role in pollen maturation and pollen–pistil interaction (Table 1). Similarly, *C.cajan_04911* (a borate transporter) that maintains boron homeostasis for pollen viability had low expression in CMS line. One gene, *C.cajan_29311*, that was downregulated in CMS line encodes cytochrome b5, which is involved in the reduction of cytochrome P‐450, a key player in flavonoid biosynthesis in the petals and seed development.

**TABLE 1:** List of some important DEGs in the present study that are reported to play important role in plant reproductive development

Gene ID	Gene annotation	*P* value	Log_2_ fold change	Regulation^a^
Carbohydrate metabolism				
*C.cajan_22752*	Malate dehydrogenase	.000256971	4.14	Up
*C.cajan_31734*	Isocitrate dehydrogenase	1.92 × 10^−5^	6.65	Up
*C.cajan_23582*	Glyceraldehyde‐3‐phosphate dehydrogenase	1.03 × 10^−10^	9.12	Up
*C.cajan_12309*	PEP carboxykinase	1.15 × 10^−8^	4.26	Up
*C.cajan_35125*	Pyruvate decarboxylase	8.30 × 10^−9^	5.08	Up
*C.cajan_00927*	Glycerol‐phosphate dehyrogenase	7.09 × 10^−12^	9.43	Up
*C.cajan_28778*	Pyruvate kinase	.000518558	−4.24	Down
Pollen development and maturation				
*C.cajan_07765*	Pollen specific protein SF3	1.24 × 10^−13^	10.13	Up
*C.cajan_19226*	L‐ascorbate oxidase	3.22 × 10^−12^	9.35	Up
*C.cajan_26704*	Pollen coat like protein	1.40 × 10^−10^	12.2	Up
*C.cajan_25431*	Olee‐1 like protein	8.03 × 10^−14^	10.25	Up
*C.cajan_41994*	Polygalactouronase	5.02 × 10^−13^	10.01	Up
*C.cajan_23980*	Expansin B3	3.55 × 10^−12^	9.68	Up
*C.cajan_10753*	Receptor like protein kinase	5.23 × 10^−9^	8.00	Up
*C.cajan_38786*	1‐Phosphatidylinositol‐4‐phosphate 5‐kinase	1.31 × 10^−5^	6.78	Up
*C.cajan_46470*	Calmodulin binding	3.96 × 10^−5^	6.39	Up
*C.cajan_02472*	Exine formation (pollen‐pistil interaction)	.000129337	6.18	Up
*C.cajan_34221*	Serine/theronine protein kinases (pollen rupturing)	1.30 × 10^−12^	9.85	Up
*C.cajan_04911*	Boron transporter	4.23 × 10^−11^	5.22	Up
Carbon fixation and photophosphorylation				
*C.cajan_42349*	PEP carboxylase	.000635824	6.06	Up
*C.cajan_46206*	RUBISCO (large subunit)	3.74 × 10^−6^	−3.80	Down
*C.cajan_28974*	PS I	.002088113	−3.80	Down
*C.cajan_28954*	Cytochrome b6	.000251454	−3.81	Down
ATPase proton pump				
*C.cajan_37897*	ATPase 9	1.95 × 10^−16^	10.83	Up
*C.cajan_21260*	Vacoular V‐type proton subunit E2	1.32 × 10^−8^	8.75	Up
*C.cajan_45656*	ATPase 6	2.79 × 10^−10^	8.42	Up
*C.cajan_29909*	ATPase 8	6.00 × 10^−14^	7.29	Up
Sucrose transporter and synthesis				
*C.cajan_19020*	Invertase	.001203719	6.13	Up
*C.cajan_35396*	Sucrose proton symporter	6.93 × 10^−17^	11.21	Up
Amino acid biosynthesis				
*C.cajan_00668*	Isoleucine, leucine, valine synthesis	1.35 × 10^−6^	7.33	Up
Pentose and glucuronate interconversions				
*C.cajan_11741*	Pectate lyases	1.59 × 10^−14^	10.32	Up

^a^ Gene expression in fertile line in comparison to cytoplasmic male sterility line.

The gene *C.cajan_19641* coding for cytochrome c oxidase assembly protein subunit 11 (COX 11) showed abundant expression in the CMS line. Cytochrome c oxidase is the marker enzyme attached to mitochondrial inner membrane (Liu et al., 2013). Early cytochrome c release, ROS burst, and abnormal tapetal PCD have been reported to cause pollen abortion in Welsh onion CMS lines (Liu et al., 2016). In this context, we also observed higher expression level of cysteine proteinase (*C.cajan_06109*) in the flower buds of fertile plants, leading to timely degeneration of tapetum and production of functional pollen grains. Phosphatidylinositol 3, 4, 5 trisphosphate 3‐phosphatase (PTEN) protein and cAMP binding protein (also known as calmodulin‐binding protein) directly involved in Ca^+2^ ions mediated signaling controlling pollen tube maturation were also found to be upregulated in fertile line.

### 3.6 Validation of the DEGs using qRT‐PCR

**FIGURE 8:**
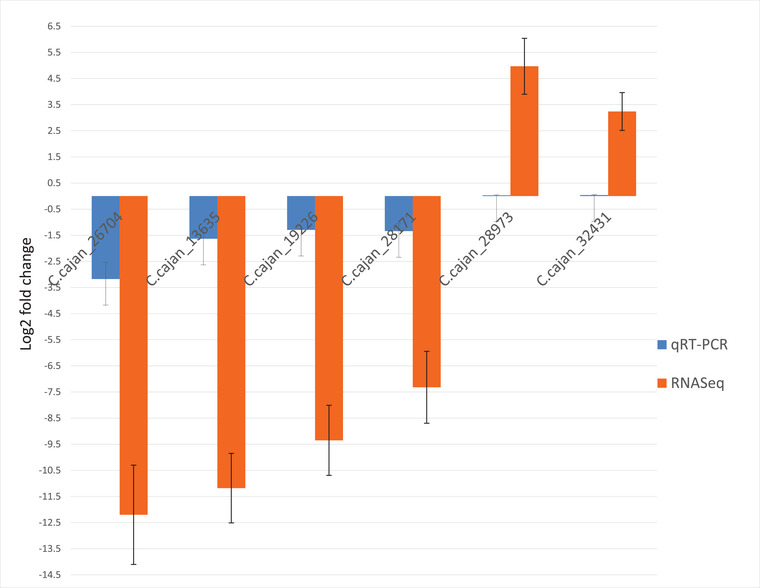
Validation of the expression of six candidate genes by quantitative real time–polymerase chain reaction. Up‐ and downregulated differentially expressed genes of the cytoplasmic male sterility line ICPL 88039A are shown

Six DEGs were chosen for qRT‐PCR in order to verify the expression patterns established from RNA‐Seq data (Table 2). The results of qRT‐PCR of the selected DEGs were highly consistent with the RNA‐seq results (Figure 8). Among these DEGs, *C.cajan_26704*, *C.cajan_13635*, *C.cajan_19226*, and *C.cajan_28973* encode pollen‐coat‐like protein, arabinogalactin protein 16, L‐ascorbate oxidase, photosystem I/P700 in chloroplast, and electron acceptor A_0_, A_1_, and F_x_, respectively. The qRT‐PCR results confirmed the RNA‐seq data. Selection of DEGs for quantitative PCR validation relied upon higher number of transcripts corresponding to a single DEG having higher fold change and possible association with the trait of interest based on literature survey.

**TABLE 2:** Details of primer pairs selected for quantitative polymerase chain reaction verification of the RNA‐sequencing data

Gene	Forward primer (5′−3′)	Tm	Reverse primer (5′−3′)	Tm	Size	Product size
		°C		°C		bp
*C.cajan_26704*	TGGACTCCCAAAACATGAGC	61	CCTTGTGCTTTTGCCTTCAT	60.2	20	154
*C.cajan_13635*	CCGTTTCCAGTGCTTCATTC	60.6	CATATGCAATCGCTTGGTCA	60.6	20	144
*C.cajan_19226*	AACAACATGCCCTATCGCTC	60.1	ATATGGGACGGGAATCAACA	60	20	163
*C.cajan_28171*	TACGAGAGCATCGTCGACAC	60	TGAGCGAAGGGATTTTCAAC	60.2	20	158
*C.cajan_28973*	TCTATTAGATCTGGCAGTGGGC	60.6	CAATCGTGAGCTACGAGCAA	60.1	20	154
*C.cajan_32431*	GTTCCGCCTTAGAGGGAAGG	62.3	TACCCCTTATTACCCCTCCG	60	20	156

## 4 DISCUSSION

Whole transcriptomic data were used to decipher molecular mechanism of CMS in the study. Anther development is a complex process involving communication between nuclear and mitochondrial genes. Four models that describe CMS occurrence in plants are energy deficiency, aberrant PCD, retrograde regulation, and cytotoxicity (Chen & Liu, 2014). The energy deficiency model associating impaired ATPase genes with the CMS trait in various crops registers concordance with our findings. ATPases, plasma membrane‐type, exhibited reduced expression in the CMS line in the current study. A similar downregulation was also observed in CMS line for the genes that participate in oxidative phosphorylation and electron transport chain like H+ transporting ATPases. Our observation finds close agreement with the transcriptome profiling of CMS and its maintainer line in Welsh onion (*Allium fistulosum* L. ) (Liu et al., 2016). According to the candidate genes reported in our study and in soybean (Li et al., 2015), carbohydrates are not only responsible to provide energy but also control pollen development as a signaling molecule and cater the higher energy demand during reproductive phase of crop plants. Rhoads et al. (2006) have suggested an increase in ROS concentration under hypoxic condition or low expression of mitochondrial complexes, influencing the normal PCD. In the present study, sucrose transporter genes *SUC 8*, *SUC 10*, and *SUC 14* showed higher expression in fertile line. Similarly, a sucrose transporter gene (*SUC 1*) suppressed by the miRNA was found in Chinese cabbage [*Brassica rapa* L. subsp. *pekinensis* (Lour.) Hanelt] ogura‐CMS sterile line (Tyms) (Wei et al., 2015). In the fertile line, we also noticed enhanced expression for the genes that are related to cell wall loosening, such as polygalactouronase, expansins, pectate lyase, beta 1–3 glucosidase, endoglucanase, which is in accordance with the findings reported earlier by Rhee et al. (2015) based on differential gene expression between the sterile line and its near isogenic fertile line. Likewise, significant differences in transcript abundance were monitored between CMS and its fertile line in soybean for genes participating in pollen wall development like plant invertase and pectin methylesterase inhibitor and pectate lyase family proteins (Li et al., 2015).

Our observations gather strong support from gene expression atlas (CcGEA) in pigeonpea (Pazhamala et al., 2017). Most importantly, the three highly connected hub genes—sucrose‐proton symporter 2 (*C.cajan_35396*), a pollen specific SF3 protein (*C.cajan_07765*), and an uncharacterized protein (*C.cajan_28171*) of the expression network for flower‐related genes—showed higher expression in the fertile line. Interestingly, expression pattern of *C.cajan_28171* was further validated by qRT‐PCR analysis in the present study. Similarly, pentose and glucuronate interconversions was represented by 18 genes in our analysis, and the genes such as *C.cajan_44741, C.cajan_04312*, and *C.cajan_27022* had lower expression in CMS lines. In CMS line, we also noted low expression of pollen‐specific genes like L‐ascorbate oxidase (*C.cajan_19226*), β–galactosidase (*C.cajan_32927*), polygalacturonase (*C.cajan_04312*), etc. Pazhamala et al. (2017) also associated pollen viability in pigeonpea with impairments in boron homeostasis, which concurs with our observation on differential expression of boron transporters (*C.cajan_04911, C.cajan_39524*) between the two lines. Consistent with earlier reports, we observed lower expression of arabinogalactan glycoproteins (AGPs), such as GPI‐anchored proteins and Fasciclin‐like arabinogalactan protein 3, in the CMS line, suggesting their participation in impaired microspore development (Rhee et al., 2015; Saxena et al., 2020).

Specific expression of *BoPMEI1*, a pollen‐specific pectin methylesterase inhibitor, in mature pollen grains and tubes confirms the participation of pectin methylesterase inhibitors as candidate genes in pollen tube growth (Zhang et al., 2010). Other genes, such as those coding for pollen Ole e 3 allergen and expansin family protein, which have been established as developmental regulators, also showed downregulation in CMS line vs. fertile line. This observation also remains in close agreement with the findings reported earlier in soybean based on DEG profiling of CMS line and its fertile counterpart (Li et al., 2015).

Enhanced expression of genes encoding late embryogenesis abundant (LEA) proteins in fertile line is also noteworthy as LEA‐like proteins, such as LP28, participate in pollen maturation in several crops [e.g., lily (*Lilium longiflorum* Thunb.)] and the presence of LP 28 in pollen tube wall implied toward its contribution in the growth of the pollen tube (Mogami et al., 2002). Importantly, the antioxidant activity of LEA protein in eliminating ROS and protecting cell wall was elucidated in soybean (Liu et al., 2011). For that reason, inhibition of ROS scavenging enzymes is considered as a potential reason for mitochondrial damage and subsequent sterility trait in soybean CMS line (Li et al., 2015).

Greater abundance of calmodulins (CaM) and calmodulin‐like (CML 11) proteins in fertile line also supported their function in pollen development. Evidence suggests a connection between CaM and signal transduction mechanisms that regulate growth and orientation of pollen tube (Rato et al., 2004). We also observed enhanced expression of Ca^++^–dependent protein kinase (CPK) in fertile line in this study. In a similar manner, prevalence of 12 of the 34 *Arabidopsis thaliana* (L.) Heynh. CPK genes was demonstrated in pollen, and importantly, *cpk17* and *cpk34* could be conclusively associated with perturbation in pollen tube growth and their Ca^++^–dependent activation was also illustrated (Myers et al., 2009). Golovkin and Reddy (2003) ruled out the role of calmodulin‐binding protein (No Pollen Germination1: NPG1) in microsporogenesis; however, their implications to pollen germination were established. Two more DEGs corresponding to calcium‐binding EF‐hand family showed upregulation in fertile line. Another DEG, relevant to plant fertility, is glutamate decarboxylase 1 (GAD), which generates gamma‐aminobutyric acid from L glutamic acid. Various research groups have generated evidences that confirm the role of gamma‐aminobutyric acid in guidance of pollen tube growth (Biancucci et al., 2015).

We also observed differential expression of genes associated with various metabolic events, in particular, glucose and lipid metabolism. Genes related to glycolysis and gluconeogenesis‐like phosphoglycerate kinase, phosphoenolpyruvate carboxykinase, and fructokinase showed downregulation in CMS line. Similarly, glycerol‐3‐phosphate dehydrogenase, connecting carbohydrate metabolism with lipid metabolism leading to the formation of glycerol and electrons in mitochondria, also showed downregulation in CMS line. Based on histological and RNA‐seq data analysis, defects in pollen wall formation resulting from disrupted fatty acid synthesis were shown to be causative for male sterility in cotton (*Gossypium* spp.) GMS line (Wu et al., 2015).

## 5 CONCLUSION

We performed high‐throughput transcriptome analysis using Illumina sequencing technology to study the differential gene expression patterns between the two isogenic alloplasmic lines of pigeonpea. Consequently, a set of 505 genes showed marked differences in their expression level between the sterile and fertile lines. Based on GO, BiNGO, and KEGG analysis we could establish a possible connection of CMS trait of pigeonpea with perturbations in glucose and lipid metabolism, energy deficiency, pollen development and pollen tube growth, and ROS generation and PCD. Our findings are congruent with a previous report in pigeonpea (Saxena et al., 2020) and similar mechanisms reported in other crops. Our findings offer a comprehensive foundation to accelerate functional genomics of CMS trait of pigeonpea. However, future research will be required to validate the specific functions of genes associated with pigeonpea CMS.

## CONFLICT OF INTEREST

The authors declare that they have no conflict of interest.

## AUTHOR CONTRIBUTIONS

AB conceptualized study and planned experiments; AB, SNSJ, SP, RJ, LP, AKM, KRS, MA, GP carried out the experiments; AB, PG, AR, performed analysis and interpretation; AB prepared the original draft with support from PG, AT and RKV; RKS, RKV, DD, NPS review and edited the manuscript. All authors have read and approved the final manuscript.

## Supporting information

Supplemental Figure S1. Principal component analysis illustrating the overall effect of experimental covariates

Supplemental Figure S2. Sample‐to‐sample distance matrix that shows relationships among three biological replicates of the genotypes ICPL 88039A and ICPL 88039B

Supplemental Figure S3. GO terms of upregulated genes associated with cellular component. Color represents the relative significance, ranging from dark red (most significant) to bright yellow (least significant). (A) GO enrichment using topGo (B) GO enrichment using BiNGO

Supplemental Figure S3b.

Supplemental Figure S4. DEGs affecting the KEGG pathway “Pentose and glucuronate interconversions”

Supplemental Figure S5. Overview of the metabolism of the DEGs by MapMan. The color key represents normalized log2 values, with red and blue representing downregulation and upregulation, respectively

Supplemental Table S1. List of all identified SNPs in ICPL 88039A and ICPL 88039B

Supplemental Table S2. List of differentially expressed transcripts/genes upregulated and downregulated in ICPL 88039B

Supplemental Table S3. Identification of enriched GO terms of the DEGs using topGO analysis

Supplemental Table S4. BiNGO‐based mapping of predominant functional themes of the DEGs

Supplemental Table S5. Identified enriched KEGG pathways from differentially expressed genes

Supplemental Table S6. List of the hub genes identified by PPI network analysis and GO annotations

## References

[tpg220132-bib-0001] Alexa, A. , & Rahnenfuhrer, J. (2019). *TopGO: Enrichment analysis for gene ontology*. R package version 2.36.0. https://rdrr.io/bioc/topGO/

[tpg220132-bib-0002] Andrews, S. (2010). FastQC: A quality control tool for high throughput sequence data . http://www.bioinformatics.babraham.ac.uk/projects/fastqc

[tpg220132-bib-0003] Biancucci, M. , Mattioli, R. , Forlani, G. , Funck, D. , Costantino, P. , & Trovato, M. (2015). Role of proline and GABA in sexual reproduction of angiosperms. Frontiers in Plant Science, 6, 680. 10.3389/fpls.2015.00680 26388884 PMC4559642

[tpg220132-bib-0004] Blighe, K. , Rana, S. , & Lewis, M. (2019). *EnhancedVolcano: Publication‐ready volcano plots with enhanced colouring and labeling*. R package version 1.4.0. https://bioconductor.org/packages/release/bioc/vignettes/EnhancedVolcano/inst/doc/EnhancedVolcano.html

[tpg220132-bib-0005] Bohra, A. , Jha, R. , Singh, I. P. , Pandey, G. , Pareek, S. , Basu, P. S. , Chaturvedi, S. K. , & Singh, N. P. (2017). Novel CMS lines in pigeonpea [*Cajanus cajan* (L.) Millspaugh] derived from cytoplasmic substitutions, and their effective restoration and deployment in hybrid breeding. The Crop Journal, 5, 89–94. 10.1016/j.cj.2016.10.003

[tpg220132-bib-0006] Bohra, A. , Jha, U. C. , Adhimoolam, P. , Bisht, D. , & Singh, N. P. (2016). Cytoplasmic male sterility (CMS) in hybrid breeding in field crops. Plant Cell Reports, 35, 967–993. 10.1007/s00299-016-1949-3 26905724

[tpg220132-bib-0007] Bohra, A. , Saxena, K. B. , Varshney, R. K. , & Saxena, R. K. (2020). Genomics‐assisted breeding for pigeonpea improvement. Theoretical and Applied Genetics, 133, 1721–1737. 10.1007/s00122-020-03563-7 32062675

[tpg220132-bib-0008] Bolger, A. M. , Lohse, M. , & Usadel, B. (2014). Trimmomatic: A flexible trimmer for Illumina sequence data. Bioinformatics, 30, 2114–2120. 10.1093/bioinformatics/btu170 24695404 PMC4103590

[tpg220132-bib-0009] Camacho, C. , Coulouris, G. , Avagyan, V. , Ma, N. , Papadopoulos, J. , Bealer, K. , & Madden, T. L. (2009). BLAST+: Architecture and applications. BMC Bioinformatics, 10, 421. 10.1186/1471-2105-10-421 20003500 PMC2803857

[tpg220132-bib-0010] Chen, L. , & Liu, Y. G. (2014). Male sterility and fertility restoration in crops. Annual Review of Plant Biology, 65, 579–606. 10.1146/annurev-arplant-050213-040119 24313845

[tpg220132-bib-0011] Das, P. , & Majumder, A. L. (2019). Transcriptome analysis of grapevine under salinity and identification of key genes responsible for salt tolerance. Functional & Integrative Genomics, 19, 61–73. 10.1007/s10142-018-0628-6 30046943

[tpg220132-bib-0012] Dobin, A. , Davis, C. A. , Schlesinger, F. , Drenkow, J. , Zaleski, C. , Jha, S. , Batut, P. , Chaisson, M. , & Gingeras, T. R. (2012). STAR: Ultrafast universal RNA‐seq aligner. Bioinformatics, 29, 15–21. 10.1093/bioinformatics/bts635 23104886 PMC3530905

[tpg220132-bib-0013] Dong, D. K. , Li, Z. , Yuan, F. J. , Zhu, S. L. , Chen, P. , Yu, W. , Yang, Q. H. , Fu, X. J. , Yu, X. M. , Li, B. Q. , & Zhu, D. H. (2012). Inheritance and fine mapping of a restorer‐of‐fertility (*Rf*) gene for the cytoplasmic male sterility in soybean. Plant Science, 188–189, 36–40. 10.1016/j.plantsci.2012.02.007 22525242

[tpg220132-bib-0014] Golovkin, M. , & Reddy, A. S. N. (2003). A calmodulin‐binding protein from Arabidopsis has an essential role in pollen germination. Proceedings of the National Academy of Sciences of the United States of America, 100, 10558–10563. 10.1073/pnas.1734110100 12928497 PMC193600

[tpg220132-bib-0015] Grabherr, M. G. , Haas, B. J. , Yassour, M. , Levin, J. Z. , Thompson, D. A. , Amit, I. , Adiconis, X. , Fan, L. , Raychowdhury, R. , Zeng, Q. , Chen, Z. , Mauceli, E. , Hacohen, N. , Gnirke, A. , Rhind, N. , Di Palma, F. , Birren, B. W. , Nusbaum, C. , Lindblad‐Toh, K. , … Regev, A. (2011). Full‐length transcriptome assembly from RNA‐Seq data without a reference genome. Nature Biotechnology, 29, 644–652. 10.1038/nbt.1883 PMC3571712 21572440

[tpg220132-bib-0016] Huang, M. D. , Hsing, Y. I. , & Huang, A. H. (2011). Transcriptomes of the anther sporophyte: Availability and uses. Plant & Cell Physiology, 52, 1459–1466. 10.1093/pcp/pcr088 21743085 PMC3172567

[tpg220132-bib-0017] Jorge, A. R. , & Omar, Z. (2011). Isolation of total RNA from tissues rich in polyphenols and polysaccharides of mangrove plants. Electronic Journal of Biotechnology, 14, 1–8.

[tpg220132-bib-0018] Krzywinski, M. , Schein, J. , Birol, I. , Connors, J. , Gascoyne, R. , Horsman, D. , Jones, S. J. , & Marra, M. A. (2009). Circos: An information aesthetic for comparative genomics. Genome Research, 19, 1639–1645. 10.1101/gr.092759.109 19541911 PMC2752132

[tpg220132-bib-0019] Langmead, B. , & Salzberg, S. L. (2012). Fast gapped‐read alignment with Bowtie 2. Nature Methods, 9, 357–359. 10.1038/nmeth.1923 22388286 PMC3322381

[tpg220132-bib-0020] Li, B. , & Dewey, C. N. (2011). RSEM: Accurate transcript quantification from RNA‐Seq data with or without a reference genome. BMC Bioinformatics, 12, 323. 10.1186/1471-2105-12-323 21816040 PMC3163565

[tpg220132-bib-0021] Li, J. , Han, S. , Ding, X. , He, T. , Dai, J. , Yang, S. , & Gai, J. (2015). Comparative transcriptome analysis between the cytoplasmic male sterile line NJCMS1A and its maintainer NJCMS1B in soybean (*Glycine max* (L.) Merr.). PLoS ONE, 10, e0126771. 10.1371/journal.pone.0126771 25985300 PMC4436259

[tpg220132-bib-0022] Liu, C. , Ma, N. , Wang, P. Y. , Fu, N. , & Shen, H. L. (2013). Transcriptome sequencing and de novo analysis of a cytoplasmic male sterile line and its near‐isogenic restorer line in chili pepper (*Capsicum annuum* L.). PLoS ONE, 8, e65209. 10.1371/journal.pone.0065209 23750245 PMC3672106

[tpg220132-bib-0023] Liu, G. , Xu, H. , Zhang, L. , & Zheng, Y. (2011). Fe binding properties of two soybean (*Glycine max* L.) LEA4 proteins associated with antioxidant activity. Plant & Cell Physiology, 52, 994–1002. 10.1093/pcp/pcr052 21531760

[tpg220132-bib-0024] Liu, Q. , Lan, Y. , Wen, C. , Zhao, H. , Wang, J. , & Wang, Y. (2016). Transcriptome sequencing analyses between the cytoplasmic male sterile line and its maintainer line in Welsh onion (*Allium fistulosum* L.). International Journal of Molecular Sciences, 17, 1058. 10.3390/ijms17071058 27376286 PMC4964434

[tpg220132-bib-0025] Livak, K. J. , & Schmittgen, T. D. (2001). Analysis of relative gene expression data using real‐time quantitative PCR and the 2^−ΔΔCT^ method. Methods, 25, 402–408. 10.1006/meth.2001.1262 11846609

[tpg220132-bib-0026] Love, M. I. , Huber, W. , & Anders, S. (2014). Moderated estimation of fold change and dispersion for RNA‐seq data with DESeq2. Genome Biology, 15, 550. 10.1186/s13059-014-0550-8 25516281 PMC4302049

[tpg220132-bib-0027] Maere, S. , Heymans, K. , & Kuiper, M. (2005). BiNGO: A Cytoscape plugin to assess overrepresentation of gene ontology categories in biological networks. Bioinformatics, 21, 3448–3449. 10.1093/bioinformatics/bti551 15972284

[tpg220132-bib-0028] McKenna, A. , Hanna, M. , Banks, E. , Sivachenko, A. , Cibulskis, K. , Kernytsky, A. , Garimella, K. , Altshuler, D. , Gabriel, S. , Daly, M. , & Depristo, M. A. (2010). The Genome Analysis Toolkit: A MapReduce framework for analyzing next‐generation DNA sequencing data. Genome Research, 20, 1297–1303. 10.1101/gr.107524.110 20644199 PMC2928508

[tpg220132-bib-0029] Mishra, A. , & Bohra, A. (2018). Non‐coding RNAs and plant male sterility: Current knowledge and future prospects. Plant Cell Reports, 37, 177–191 10.1007/s00299-018-2248-y 29332167

[tpg220132-bib-0030] Mogami, N. , Shiota, H. , & Tanaka, I. (2002). The identification of a pollen‐specific LEA‐like protein in *Lilium longiflorum* . Plant Cell Environment, 25, 653–663. 10.1046/j.1365-3040.2002.00852.x

[tpg220132-bib-0031] Myers, C. , Romanowsky, S. M. , Barron, Y. D. , Garg, S. , Azuse, C. L. , Curran, A. , Davis, R. M. , Hatton, J. , Harmon, A. C. , & Harper, J. F. (2009). Calcium‐dependent protein kinases regulate polarized tip growth in pollen tubes. The Plant Journal, 59, 528–539. 10.1111/j.1365-313X.2009.03894.x 19392698

[tpg220132-bib-0032] Pazhamala, L. T. , Purohit, S. , Saxena, R. K. , Garg, V. , Krishnamurthy, L. , Verdier, J. , & Varshney, R. K. (2017). Gene expression atlas of pigeonpea and its application to gain insights into genes associated with pollen fertility implicated in seed formation. Journal of Experimental Botany, 68, 2037–2054. 10.1093/jxb/erx010 28338822 PMC5429002

[tpg220132-bib-0033] Rato, C. , Monteiro, D. , Hepler, P. K. , & Malhó, R. (2004). Calmodulin activity and cAMP signalling modulate growth and apical secretion in pollen tubes. The Plant Journal, 38, 887–897. 10.1111/j.1365-313X.2004.02091.x 15165182

[tpg220132-bib-0034] Rhee, S. J. , Seo, M. , Jang, Y. J. , Cho, S. , & Lee, G. P. (2015). Transcriptome profiling of differentially expressed genes in floral buds and flowers of male sterile and fertile lines in watermelon. BMC Genomics, 16, 914. 10.1186/s12864-015-2186-9 26552448 PMC4640349

[tpg220132-bib-0035] Rhoads, D. M. , Umbach, A. L. , Subbaiah, C. C. , & Siedow, J. N. (2006). Mitochondrial reactive oxygen species. Contribution to oxidative stress and interorganellar signaling. Plant Physiology, 141, 357–366. 10.1104/pp.106.079129 16760488 PMC1475474

[tpg220132-bib-0036] Saxena, K. B. , Kumar, R. V. , Tikle, A. N. , Saxena, M. K. , Gautam, V. S. , Rao, S. K. , Khare, D. K. , Chauhan, Y. S. , Saxena, R. K. , Reddy, B. V. S. , Sharma, D. , Reddy, L. J. , Green, J. M. , Faris, D. G. , Nene, Y. L. , Mula, M. , Sultana, R. , Srivastava, R. K. , Gowda, C. L. L. , … Varshney, R. K. (2013). ICPH 2671—The world's first commercial food legume hybrid. Plant Breeding, 132, 479–485. 10.1111/pbr.12045

[tpg220132-bib-0037] Saxena, K. B. , Sultana, R. , Mallikarjuna, N. , Saxena, R. K. , Kumar, R. V. , Sawargaonkar, S. L. , & Varshney, R. K. (2010). Male‐sterility systems in pigeonpea and their role in enhancing yield. Plant Breeding, 129, 125–134. 10.1111/j.1439-0523.2009.01752.x

[tpg220132-bib-0038] Saxena, S. , Sahu, S. , Kaila, T. , Nigam, D. , Chaduvla, P. K. , Rao, A. R. , Sanand, S. , Singh, N. K. , & Gaikwad, K. (2020). Transcriptome profiling of differentially expressed genes in cytoplasmic male‐sterile line and its fertility restorer line in pigeon pea (*Cajanus cajan* L.). BMC Plant Biology, 20, 74. 10.1186/s12870-020-2284-y 32054447 PMC7020380

[tpg220132-bib-0039] Schwacke, R. , Ponce‐Soto, G. Y. , Krause, K. , Bolger, A. M. , Arsova, B. , Hallab, A. , Gruden, K. , Stitt, M. , Bolger, M. E. , & Usadel, B. (2019). MapMan4: A refined protein classification and annotation framework applicable to multi‐omics data analysis. Molecular Plant, 12, 879–892. 10.1016/j.molp.2019.01.003 30639314

[tpg220132-bib-0040] Shannon, P. (2003). Cytoscape: A software environment for integrated models of biomolecular interaction networks. Genome Research, 13, 2498–2504. 10.1101/gr.1239303 14597658 PMC403769

[tpg220132-bib-0041] Sinha, P. , Saxena, K. B. , Saxena, R. K. , Singh, V. K. , Suryanarayana, V. , Sameer Kumar, C. V. , Katta, M. A. V. S. , Khan, A. W. , & Varshney, R. K. (2015a). Association of *nad7a* gene with cytoplasmic male sterility in pigeonpea. The Plant Genome, 8. 10.3835/plantgenome2014.11.0084 33228303

[tpg220132-bib-0042] Sinha, P. , Singh, V. K. , Suryanarayana, V. , Krishnamurthy, L. , Saxena, R. K. , & Varshney, R. K. (2015b). Evaluation and validation of housekeeping genes as reference for gene expression studies in pigeonpea (*Cajanus cajan*) under drought stress conditions. PLoS ONE, 10, e0122847. 10.1371/journal.pone.0122847 25849964 PMC4388706

[tpg220132-bib-0043] Szklarczyk, D. , Gable, A. L. , Lyon, D. , Junge, A. , Wyder, S. , Huerta‐Cepas, J. , Simonovic, M. , Doncheva, N. T. , Morris, J. H. , Bork, P. , Jensen, L. J. , & Mering, C. . V. (2018). STRING v11: Protein–protein association networks with increased coverage, supporting functional discovery in genome‐wide experimental datasets. Nucleic Acids Research, 47, D607–D613. 10.1093/nar/gky1131 PMC6323986 30476243

[tpg220132-bib-0044] Tuteja, R. , Saxena, R. K. , Davila, J. , Shah, T. , Chen, W. , Xiao, Y. L. , Fan, G. , Saxena, K. B. , Alverson, A. J. , Spillane, C. , Town, C. , & Varshney, R. K. (2013). Cytoplasmic male sterility‐associated chimeric open reading frames identified by mitochondrial genome sequencing of four *Cajanus* genotypes. DNA Research, 20, 485–495. 10.1093/dnares/dst025 23792890 PMC3789559

[tpg220132-bib-0045] Wei, X. , Zhang, X. , Yao, Q. , Yuan, Y. , Li, X. , Wei, F. , Zhao, Y. , Zhang, Q. , Wang, Z. , Jiang, W. , & Zhang, X. (2015). The miRNAs and their regulatory networks responsible for pollen abortion in Ogura‐CMS Chinese cabbage revealed by high‐throughput sequencing of miRNAs, degradomes, and transcriptomes. Frontiers in Plant Science, 6, 894. 10.3389/fpls.2015.00894 26557132 PMC4617173

[tpg220132-bib-0046] Wu, Y. , Min, L. , Wu, Z. , Yang, L. , Zhu, L. , Yang, X. , Yuan, D. , Guo, X. , & Zhang, X. (2015). Defective pollen wall contributes to male sterility in the male sterile line 1355A of cotton. Scientific Reports, 5, 10. 10.1038/srep09608 PMC4456728 26043720

[tpg220132-bib-0047] Xie, C. , Mao, X. , Huang, J. , Ding, Y. , Wu, J. , Dong, S. , Kong, L. , Gao, G. , Li, C. Y. , & Wei, L. (2011). KOBAS 2.0: A web server for annotation and identification of enriched pathways and diseases. Nucleic Acids Research, 39, W316–W322. 10.1093/nar/gkr483 21715386 PMC3125809

[tpg220132-bib-0048] Yang, P. , Han, J. , & Huang, J. (2014). Transcriptome sequencing and de novo analysis of cytoplasmic male sterility and maintenance in JA‐CMS cotton. PLoS ONE, 9, e112320. 10.1371/journal.pone.0112320 25372034 PMC4221291

[tpg220132-bib-0049] Zhang, G. Y. , Feng, J. , Wu, J. , & Wang, X. W. (2010). BoPMEI1, a pollen‐specific pectin methylesterase inhibitor, has an essential role in pollen tube growth. Planta, 231, 1323–1334. 10.1007/s00425-010-1136-7 20229192

